# Miniaturized Fluorimetric Method for Quantification of Zinc in Dry Dog Food

**DOI:** 10.1155/2020/8821809

**Published:** 2020-09-04

**Authors:** Rute C. Martins, Ana M. Pereira, Elisabete Matos, Luisa Barreiros, António J. M. Fonseca, Ana R. J. Cabrita, Marcela A. Segundo

**Affiliations:** ^1^LAQV, REQUIMTE, Departamento de Ciências Químicas, Faculdade de Farmácia, Universidade do Porto, Rua Jorge Viterbo Ferreira no 228, Porto 4050-313, Portugal; ^2^LAQV, REQUIMTE, Instituto de Ciências Biomédicas de Abel Salazar (ICBAS), Universidade do Porto, Rua de Jorge Viterbo Ferreira no 228, Porto 4050-313, Portugal; ^3^SORGAL, Sociedade de Óleos e Rações S.A., Estrada Nacional 109 Lugar da Pardala, São João 3880-728, Ovar, Portugal

## Abstract

Zinc is an essential trace element for animals in several biological processes, particularly in energy production, and it is acquired from food ingestion. In this context, a microplate-based fluorimetric assay was developed for simple, fast, and low-cost determination of zinc in pet food using 2,2′-((4-(2,7-difluoro-3,6-dihydroxy-4a*H*-xanthen-9-yl)-3-methoxyphenyl)azanediyl)diacetic acid (FluoZin-1) as fluorescent probe. Several aspects were studied, namely, the stability of the fluorescent product over time, the FluoZin-1 concentration, and the pH of reaction media. The developed methodology provided a limit of detection of 1 *μ*g L^−1^ in sample acid digests, with a working range of 10 to 200 *μ*g L^−1^, corresponding to 100–2000 mg of Zn per kg of dry dog food samples. Intraday repeatability and interday repeatability were assessed, with relative standard deviation values < 3.4% (100 *μ*g L^−1^) and <11.7% (10 *μ*g L^−1^). Sample analysis indicated that the proposed fluorimetric assay provided results consistent with ICP-MS analysis. These results demonstrated that the developed assay can be used for rapid determination of zinc in dry dog food.

## 1. Introduction

Zinc is an important element in the feeding of living beings, as it intervenes in the metabolism of proteins and nucleic acids, as well as in the appropriate functioning of biological systems. It exists in the whole body, as zinc(II) or Zn^2+^, mainly as an intracellular constituent, and it is present in most tissues at relatively low concentrations [1]. Concerning the existence in dogs, total body Zn content of puppies and young adult dogs is on average 23.1 and 9.5 mg·kg^−1^ BW (body weight), respectively, and it is acquired through the ingested food [2].

The absorption of dietary zinc might be affected by other dietary constituents, which alters its bioavailability from food. The absorption of zinc can be disturbed by compounds from the vegetable ingredients present in dog food, among which phytate assumes particular importance. In fact, dietary phytate reduces the absorption of zinc, and this effect is aggravated by high concentrations of edible calcium. Furthermore, the zinc deficiency is commonly correlated with growth retardation in young animals [3]. Zinc is an almost nontoxic substance, but it can sometimes become toxic when it interacts with other nutrients in the animal's body. A few cases of inadvertent overconsumption of zinc by dogs, namely, by puppies, have been reported, and the clinical signs detected included acute gastroenteritis and anemia [3]. For zinc in particular, the minimum recommended level in complete food for adult dogs is 7.20 to 8.34 mg per 100 g of dry matter, with a higher level for puppies (10.00 mg per 100 g of dry matter) [4]. There is also a maximum legal limit established in the EU, corresponding to 22.7 mg per 100 g of dry matter. [5].

The need for quality control, particularly during formulation of commercial dry dog food, requires fast and simple methods for zinc determination. To date, various methods have been proposed to determine zinc, namely, conventional methods based on atomic features, including inductively coupled plasma mass spectrometry (ICP-MS) [6, 7], atomic emission spectrometry (ICP-AES) [8], atomic absorption spectrophotometry (AAS) [9], and energy dispersive X-ray fluorescence (ED-XRF) [10]. Nevertheless, these methods require trained personnel and high-cost/high maintenance equipment. Thus, there are some rapid and low-cost methods for zinc determination, based on fluorimetric [11] or colorimetric assays [12, 13] and on near-infrared reflectance spectroscopy [14].

Several fluorimetric probes are available for assessment of zinc [15], particularly tailored for zinc assay in living cells using microscopy. FluoZin-1 (2,2′-((4-(2,7-difluoro-3,6-dihydroxy-4a*H*-xanthen-9-yl)-3-methoxyphenyl)azanediyl)diacetic acid), for instance, has been initially proposed for intracellular Zn^2+^ imaging and quantitation in the low hundred nanomolar concentration range, as it presents a large dynamic range. Other applications of this probe include the determination of stability constants of Cd(II) and Zn(II) complexes with thiols [16], the estimation of the apparent dissociation constant of bovine serum albumin for Zn^2+^ binding [17], and the evaluation of proton-dependent transport of Zn^2+^ mediated by integral synaptic vesicle protein SV31 in a proteoliposome model [18].

In this context, miniaturization of (bio)chemical reactions and analytical methods is a current trend, using either microchips [19, 20] or downscaling under microplate format [21, 22], fostering greener methodologies that originate less effluent, consume fewer reagents, and have a lower environmental footprint. Taking into account the need to develop a simple, fast, sensitive, and precise methodology for zinc determination to support pet food formulation using different sources of bioavailable zinc, the objective of this work is to implement a fluorimetric methodology based on FluoZin-1, aiming for the determination of zinc in pet food samples after acid digestion.

## 2. Materials and Methods

### 2.1. Chemicals and Solutions

All chemicals used in this work were of analytical reagent grade with no further purification and purchased from Sigma-Aldrich (St. Louis MO, USA) unless otherwise stated. Ultrapure water (18.2 MΩ cm) used in all experiments was obtained from a Sartorius Arium® water purification system (Goettingen, Germany). Sample digestion was performed using high-purity HNO_3_ (≥69% (w/w), TraceSELECT® (Fluka, Seelze, Germany)), and H_2_O_2_ (30% (v/v), TraceSELECT® Fluka) of p.a. grade. All plasticware used in the sample digestion and elemental analysis was immersed for, at least, 24 h in a 10% (v/v) HNO_3_ solution to ensure decontamination and then rinsed with ultrapure water.

**Figure 1:**
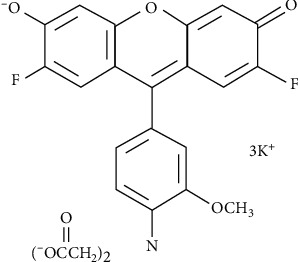
Structure of 2,2′-((4-(2, 7-difluoro-3,6-dihydroxy-4a*H*-xanthen-9-yl)-3-methoxyphenyl)azanediyl)diacetic acid (FluoZin-1).

The fluorescent reagent FluoZin-1 (2,2′-((4-(2,7-difluoro-3,6-dihydroxy-4a*H*-xanthen-9-yl)-3-methoxyphenyl)azanediyl)diacetic acid, Figure 1), tripotassium salt, cell impermeant (catalog number F24180) was acquired from Invitrogen-Thermo Fisher Scientific (Massachusetts, USA). Stock standard solutions of FluoZin-1 were prepared by dissolving the solid in 1 mL dimethyl sulfoxide (DMSO) from Merck (Darmstadt, Germany), resulting in a 0.5 mg·mL^−1^ probe solution that was stored at −20°C (with fluorescence stability during six months). Intermediate solutions of 25.0, 5.0, 2.5, and 1.25 *μ*M were prepared prior to use using either buffer or ultrapure water, depending on the experimental work performed.

The standard stock solution of Zn(II) (1.00 mg·mL^−1^) in 4% nitric acid was obtained by SCP Science (item 140-001-301, 125 mL, UN Code 3264, Baie-d'Urfé, Canada). Moreover, the stock solution of nitric acid 10 mM, used to prepare the zinc(II) standard working solutions, was prepared by diluting commercial nitric acid 70% (w w^−1^).

Several buffer solutions were used, namely, acetic acid/sodium acetate (1000 mM, pH 3.9), potassium phosphate dibasic/potassium phosphate monobasic (1000 mM, pH 7.0), and sodium hydrogen carbonate/sodium carbonate (1000 mM, pH 10.0). The working buffer solution (potassium phosphate dibasic/potassium phosphate monobasic, pH 7.0) at 5 mM was prepared by 1 : 20 dilution from a 100 mM buffer solution.

### 2.2. Fluorescence Measurements and Microplate Protocol

Fluorescence measurements were carried out using a Cytation3® microplate reader (Bio-Tek Instruments, Winooski, USA), controlled by the Gen5 (Bio-Tek Instruments) software. Solutions were placed in 96-well microplates, suitable for fluorescence measurements (black, opaque wells, ref. 10588885, Thermo Fisher Scientific, Massachusetts, USA). The excitation wavelength was set to 484 nm while the emission wavelength was 520 nm.

The final volume of the reaction mixture was set to 260 *μ*L and the assay procedure comprised the sequential addition of 160 *μ*L of phosphate buffer solution (5 mM, pH 7.0), 80 *μ*L of zinc(II) standard solution, and 20 *μ*L of FluoZin-1 solution to each well. For sample analysis, the protocol was adapted, comprising the addition of 160 *μ*L of water, 80 *μ*L of zinc(II) standard solution or diluted sample digest, and 20 *μ*L of FluoZin-1 prepared in phosphate buffer solution. Control experiments were performed by replacing each solution by their respective solvent.

A conventional fluorimeter (Model FP-6500, JASCO, Easton, USA) was applied to evaluate the excitation and emission spectra in initial studies.

### 2.3. Analysis of Pet Food Samples

Dry dog food samples were acquired in local supermarkets. Each sample was digested in duplicate, after a digestion pretreatment procedure, where the samples were oven-dried at 65°C to constant weight and then ground in a 1 mm sieve mill. Ground samples (*ca*. 500 mg) were solubilized by microwave-assisted acid digestion as described by Pereira et al. [7], using an MLS 1200 Mega high-performance microwave digestion unit (Milestone, Sorisole, Italy) equipped with an HPR-1000/10 S rotor. After weighing the sample using a plastic spatula, 3 mL of HNO_3_ and 1 mL of H_2_O_2_ were added to each polytetrafluoroethylene digestion vessel. The samples were subsequently submitted to a microwave heating program of 250 W for 1 min, 0 W for 1 min, 250 W for 5 min, 400 W for 5 min, and, finally, 650 W for 5 min. The vessels were then allowed to cool to room temperature. Thereafter, the content was transferred to 25 mL polypropylene volumetric flasks and water was added to bring up to total volume. This digest was analyzed by the developed fluorimetric assay. A blank constituted by 500 *μ*L of water was included in each digestion run.

For comparison purposes, zinc present in sample digests was also determined by inductively coupled plasma mass spectrometry (ICP-MS) using an iCAP Q™ (Thermo Fisher Scientific, Schwerte, Germany) instrument, equipped with a MicroMist™ nebulizer, a Peltier cooled cyclonic spray chamber, a standard quartz torch, and nickel skimmer and sampling cones. High-purity (99.9997%) Ar (Gasin II, Leça da Palmeira, Portugal) was used as the nebulizer and plasma gas. The ICP-MS operated under the following conditions: RF power 1550 W; auxiliary Ar flow rate 0.80 L min^−1^; nebulizer flow rate 1.08 L min^−1^, and plasma flow rate 14 L min^−1^. Zinc was determined as ^66^Zn isotopes as described in detail elsewhere [23].

## 3. Results and Discussion

### 3.1. Implementation of Fluorescence Reaction under Microplate Format

Initially, the excitation and emission spectrum of FluoZin-1 probe was evaluated using a conventional spectrofluorimeter. An increase of fluorescence intensity was observed in the presence of zinc(II), compared to the probe self-fluorescence, in the emission wavelength range 510 to 530 nm. For the excitation spectra, fluorescence intensity increased for higher wavelengths, showing a plateau at 460–470 nm, and further increased up to 490 nm. In fact, the wavelengths corresponding to the maximum fluorescence intensity were 495 and 517 nm for excitation and emission, respectively.

**Figure 2:**
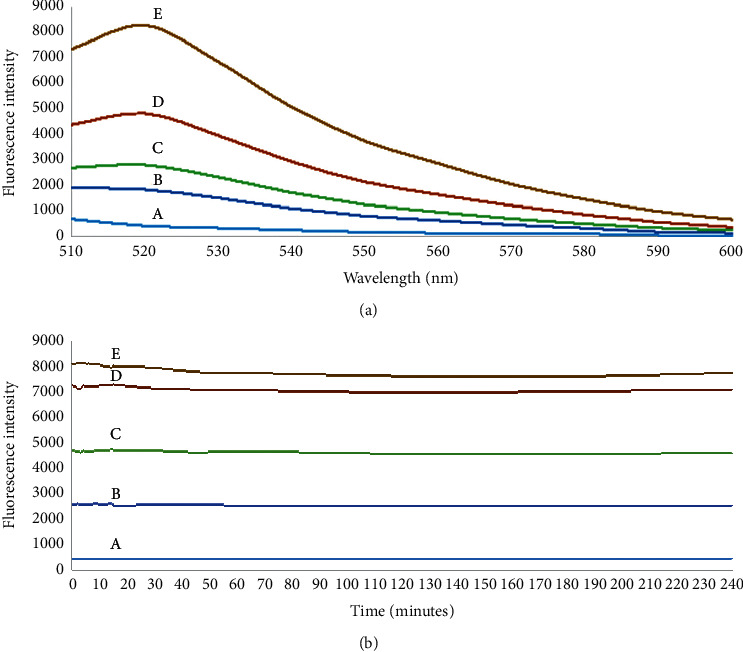
(a) FluoZin-1 probe (1.25 *μ*M) emission spectra (*λ*exc = 495 nm) with different concentrations of zinc(II). (b) Fluorescence intensity along time, using FluoZin-1 probe (1.25 *μ*M) and different concentrations of zinc(II) (*μ*g L^−1^): (A) 0, (B) 100, (C) 200, (D) 500, (E) 1000.

To transpose the detection of Zn(II)-FluoZin-1 to the current microplate equipment, a difference of at least 30 nm between excitation and emission wavelength is recommended. Therefore, the excitation wavelength was set to 484 nm while the emission wavelength was 520 nm, corresponding to the maximum emission observed in the microplate reader (Figure 2(a)).

The implementation of fluorimetric assay under microplate format required a careful choice of employed volumes. First, the volume of buffer solution was fixed at 160 *μ*L, accounting for at least 50% of the total volume and ensuring suitable pH adjustment. For standard/sample, the volume chosen was 80 *μ*L, as the expected values in pet food acid digest would be in the *μ*g per L range, providing an adequate sensitivity. Finally, considering the cost of the fluorescence probe, a lower volume was chosen (20 *μ*L), still high enough to be handled by conventional micropipettes.

Next, the effect of the FluoZin-1 concentration was evaluated. For the three tested concentrations (1.25, 2.5, and 5.0 *μ*M), there was an increase in fluorescence intensity with FluoZin-1 concentration, causing also an increase of fluorescence background (277 ± 9, 546 ± 6, and 1186 ± 44) when increasing FluoZin-1 concentration. An increase in fluorescence intensity proportional to the amount of zinc(II) present in the reaction medium was also observed (tested Zn(II) concentrations of 50–1000 *μ*g L^−1^). The sensitivity was higher for increasing concentrations of FluoZin-1, with slope values of 5.46 ± 0.29, 11.4 ± 0.5, and 22.6 ± 0.9 L·*μ*g^−1^, respectively. Further experiments were performed using 2.5 *μ*M as a compromise between sensitivity and analysis cost.

Using these conditions, the fluorescence stability over time was evaluated (Figure 2(b)). For all zinc(II) concentrations, the fluorescence was stable during 4 h, showing values corresponding to 95.6 to 98.3% in relation to the initial fluorescence intensity. Moreover, the fluorescence from FluoZin-1 in the absence of Zn(II) was also constant (Figure 2(b), A), showing no sign of photobleaching.

**Figure 3:**
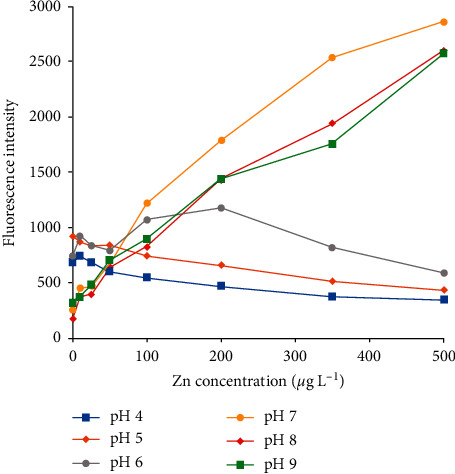
Fluorescence intensity of FluoZin-1 probe at different pH values (phosphate buffer).

Subsequently, the influence of pH of reaction media on fluorescence value was studied (Figure 3). FluoZin-1 probe was not effective for Zn(II) determination at acidic pH as the attained fluorescence was constant or even decreased in the presence of increasing zinc(II) concentrations. This can be explained by the fact that the carboxylic groups are ionized for pH values > 4, but the hydroxyl group on the unsaturated ring system presents a pKa value of 5.4, which can explain the deactivation of fluorescence observed for pH values < 6.0. For reaction media at pH 7.0 and pH 8.0, an adequate response concerning a direct relation between fluorescence and Zn(II) concentration was observed because both carboxylic groups and the hydroxyl group are ionized. In relation to reaction media at pH 9.0, a proportional response was also observed, but a decrease in the fluorescence intensity occurred for Zn(II) concentrations above 500 *μ*g L^−1^ (data not shown). Therefore, further experiments were performed at pH 7.0.

### 3.2. Analytical Features

Calibration curves were linear from 10 to 200 *μ*g L^−1^, with a typical calibration curve of *y* = 24.5 (±0.2) *x* + 1027 ± 21, *R* = 0.9993, where *y* is the fluorescence intensity and *x* is the concentration of Zn(II) expressed in *μ*g L^−1^. This working range corresponds to 100–2000 mg of Zn per kg^−1^ of dry dog food samples considering the use of 0.5 g of sample and the digestion procedure described above. The limits of detection and quantification were 1 and 9 *μ*g L^−1^, respectively, estimated by interpolation of the blank signal (*n* = 9) plus 3 or 10 times its standard deviation [24]. Accuracy and repeatability data are presented in Table 1. Accuracy was evaluated as the percentage ratio between the calculated and nominal concentrations, with acceptable values between 96.1 and 100.2%. Intraday and interday precision values were expressed as relative standard deviations (RSD%) [24], with values < 3.4% for Zn(II) concentration > 100 *μ*g L^−1^ and <11.7% for Zn(II) concentration of 10 *μ*g L^−1^.

### 3.3. Analysis of Pet Food Samples

After establishing the reaction conditions for Zn(II) determination using FluoZin-1 probe, samples were analyzed after microwave-assisted acid digestion. Initially, a digested sample was diluted 1 : 1 in water and processed by the established protocol. The fluorescence intensity was similar to that presented in the absence of zinc and remained the same after fortification with Zn(II) at 500 *μ*g L^−1^. In order to investigate if this effect was due to sample pH, digested samples and a digested blank were analyzed after dilution, with an equivalent content in nitric acid of 1.5, 3.0, and 6.0 mM. The two most acidic solutions did not provide different results among the samples and blank while, for the less acidic solutions (1.5 mM of HNO_3_), the samples presented higher fluorescence when compared to the blank and this difference was increased upon fortification with Zn(II) at 200 *μ*g L^−1^. Sample pH was an issue and the final procedure for sample analysis included a step for pH adjustment to 3.6 (and dilution 1 : 200) before analysis. Therefore, the protocol was adapted and 160 *μ*L of water was used instead of 160 *μ*L of buffer solution. As the FluoZin-1 probe solution was prepared in phosphate buffer pH 7.0, it assured that the reaction medium was at a pH value adequate for fluorescence measurements.

Sample digests were analyzed by the proposed method and by ICP-MS, providing the values reported in Table 2, expressed as mg of Zn per kg of dry food. Good agreement was obtained between the two methodologies, with relative deviations between -7.4 and 15.8%. A paired *t*-test was performed on the data obtained for these samples and a *t* value of 0.417 was calculated, which was compared to the tabulated *t* (2.144, *P*=0.05, df = 14), indicating no significant difference for the mean concentration obtained by the two methods [25]. The estimated cost of analysis per microplate was 2.70 €, accounting for a value of 0.028 € per determination.

Compared to a recent work using a microplate colorimetric assay [13], the strategy proposed here is more straightforward as it does not require the inclusion of ion masking reagents or the application of partitioning correction calculations. Compared to the fast method proposed by Goi et al. [14], our approach provides the value of the total Zn as a digestion process is performed before the fluorimetric reaction while the near-infrared reflectance spectroscopy-based method can only detect Zn associated with organic complexes, providing underestimated results for dog food supplemented with inorganic forms of Zn. Finally, compared to other approaches that applied AAS [26] or ICP-MS [7], the method proposed here allows the simultaneous determination of up to 96 samples, without requiring expensive equipment or application of the standard addition method.

## 4. Conclusions

A low-cost, environmentally friendly method for determination of zinc in dry dog food samples was developed, resorting to an innovative application of FluoZin-1. The proposed microplate format fostered the use of low amounts of reagents (<0.1 nmol of fluorescent probe), also generating a small amount (260 *μ*L) of effluent per assay. Moreover, high-throughput analysis was implemented, allowing for the simultaneous reading of up to 96 samples simultaneously, without using high maintenance/high-cost equipment based on AAS or ICP-MS. The application of the proposed methodology to the fast evaluation of zinc content during pet food formulation is envisioned.

## Figures and Tables

**Table 1:** Accuracy and precision using the conditions established for the analysis of Zn(II) in pet food.

	Intraday		Interday	
[Zn(II)] (*μ*g L^−1^)	Back-calculated concentration (%)	RSD (%)	Back-calculated concentration (%)	RSD (%)
10	100.2	11.7	107.1	9.2
100	96.1	2.0	95.0	1.7
200	98.7	1.0	96.4	3.4

**Table 2:** Zn(II) amount in pet food samples expressed as mg of Zn per kg of dry food.

Sample	Proposed method	ICP-MS method	Absolute deviation	Rd (%)
A	393 ± 14	357 ± 15	36	10.2
B	472 ± 13	421 ± 30	51	12.1
C	336 ± 10	342 ± 32	−6	−1.8
D	433 ± 25	378 ± 22	55	14.6
E	279 ± 20	286 ± 10	−7	−2.6
F	297 ± 43	256 ± 1	41	15.8
G	225 ± 9	243 ± 3	−18	−7.4
H	228 ± 19	243 ± 2	−15	−6.2

## Data Availability

Data are available upon request to the corresponding author.
